# Arsenic on the Hands of Children after Playing in Playgrounds

**DOI:** 10.1289/ehp.7197

**Published:** 2004-06-17

**Authors:** Elena Kwon, Hongquan Zhang, Zhongwen Wang, Gian S. Jhangri, Xiufen Lu, Nelson Fok, Stephan Gabos, Xing-Fang Li, X. Chris Le

**Affiliations:** ^1^Department of Public Health Sciences, University of Alberta, Edmonton, Alberta, Canada; ^2^Environmental Health, Capital Health, Edmonton, Alberta, Canada; ^3^Health Surveillance Branch, Alberta Health and Wellness, Edmonton, Alberta, Canada

**Keywords:** arsenic, CCA, children’s exposure, chromated copper arsenate, playgrounds, treated wood

## Abstract

Increasing concerns over the use of wood treated with chromated copper arsenate (CCA) in playground structures arise from potential exposure to arsenic of children playing in these playgrounds. Limited data from previous studies analyzing arsenic levels in sand samples collected from CCA playgrounds are inconsistent and cannot be directly translated to the amount of children’s exposure to arsenic. The objective of this study was to determine the quantitative amounts of arsenic on the hands of children in contact with CCA-treated wood structures or sand in playgrounds. We compared arsenic levels on the hands of 66 children playing in eight CCA playgrounds with levels of arsenic found on the hands of 64 children playing in another eight playgrounds not constructed with CCA-treated wood. The children’s age and duration of playtime were recorded at each playground. After play, children’s hands were washed in a bag containing 150 mL of deionized water. Arsenic levels in the hand-washing water were quantified by inductively coupled plasma mass spectrometry. Our results show that the ages of the children sampled and the duration of play in the playgrounds were similar between the groups of CCA and non-CCA playgrounds. The mean amount of water-soluble arsenic on children’s hands from CCA playgrounds was 0.50 μg (range, 0.0078–3.5 μg). This was significantly higher (*p* < 0.001) than the mean amount of water-soluble arsenic on children’s hands from non-CCA playgrounds, which was 0.095 μg (range, 0.011–0.41 μg). There was no significant difference in the amount of sand on the children’s hands and the concentration of arsenic in the sand between the CCA and non-CCA groups. The higher values of arsenic on the hands of children playing in the CCA playgrounds are probably due to direct contact with CCA-treated wood. Washing hands after play would reduce the levels of potential exposure because most of the arsenic on children’s hands was washed off with water. The maximum amount of arsenic on children’s hands from the entire group of study participants was < 4 μg, which is lower than the average daily intake of arsenic from water and food.

Chromated copper arsenate (CCA) is a common wood preservative (American Wood Preservers Institute 2001; Bull 2000, 2001; CPSC 2002). Starting 1 January 2004, the U.S. Environmental Protection Agency (EPA) has banned CCA as a preservative for wood intended for residential use. Despite the ban in the United States, the CCA-treated wood is used in many existing structures in residential decks and public playgrounds. Approximately 70% of single-family homes in the United States have decks and porches containing CCA, and 14% of public playgrounds have CCA wood structures (U.S. EPA 2002a, 2003a, 2003b, 2004). In Edmonton, Alberta, Canada, at the time of this study, 222 of 316 city-owned public playgrounds were constructed either totally or partially with CCA-treated wood.

Although there are numerous studies on arsenic dislodging and leaching from CCA-treated wood [Cooper 1990, 1991; Cooper et al. 2001; Henningsson and Carlsson 1984; Hingston et al. 2001, 2002; Stilwell and Gorny 1997; Stilwell et al. 2003; Taylor et al. 2001; Townsend et al. 2003; U.S. Department of Agriculture (USDA) 1996], there is little quantitative information on arsenic exposure due to CCA-treated wood in playgrounds. Studies on CCA-treated wood have mostly examined soil and sand samples from playgrounds (Balasoiu et al. 2001; Stilwell and Gorny 1997; Townsend et al. 2003; Zagury et al. 2003). None of these studies have measured the levels of arsenic found on the hands of children playing on the CCA-treated structures. Thus, risk assessment has relied on estimates of exposure assessment with many assumptions [Consumer Product Safety Commission (CPSC) 1990; Hemond and Solo-Gabriele 2004; U.S. EPA 2001, 2002b]. In the absence of more reliable measured values of arsenic actually found on children’s hands, predictions were made using calculations of average child hand surface area, adherence factors for various types of soil, and activity patterns and behaviors of children at playgrounds (Hemond and Solo-Gabriele 2004; U.S. EPA 2001 U.S. EPA 2002b). To improve exposure assessment of arsenic from playgrounds, it is essential to obtain information on the levels of arsenic on children’s hands. However, there is no direct measurement of arsenic levels on the hands of children in contact with either CCA-treated wood or the soil/sand. The purpose of this project is to fill this gap.

Our objective in this study was to determine the quantitative amount of arsenic on the hands of children in contact with CCA-treated wood structures and sand in playgrounds. We chose eight playgrounds that were constructed with CCA-treated wood and another eight playgrounds that did not contain CCA-treated wood. After play, the children’s hands were washed and the arsenic concentration on the hands and in the sand was measured in the washing. Arsenic levels on the hands of 66 children playing in CCA-treated playgrounds are compared with levels of arsenic found on the hands of 64 children playing in playgrounds that are not constructed with CCA-treated wood. This information on the level of arsenic exposure is essential to a reliable assessment of the health risks to the public with the existence of CCA-treated wood structures in playgrounds.

## Materials and Methods

### Playground selection.

The city of Edmonton owned and operated 316 public playgrounds at the time of this study, of which 222 were constructed either totally or partially with CCA-treated wood. For this study, we selected 16 playgrounds constructed between 1985 and 2003. Eight playgrounds contained CCA-treated wood structures and the other eight did not. We selected the playgrounds to represent various characteristics of the playgrounds in the city. In particular, the age of playgrounds, the manufacturers, and the geographic locations of the playgrounds in the city were similar between the CCA and non-CCA groups.

### Children’s hand-washing samples.

During 5–21 August 2003, the 16 playgrounds were visited for sampling in a randomized order, with CCA and non-CCA playgrounds interspersed throughout the sampling period. Weather conditions as well as the date and time of arrival were recorded for every playground visited. Except for damp conditions recorded for three CCA playgrounds (C, D, and N) and two non-CCA playgrounds (E and H) because of light rainfalls during the previous night, dry conditions were recorded for all other sampling days.

The time that children arrived at each playground was recorded. Parents of the children were asked for permission to allow their children’s participation in the study. The study objectives, procedures, and potential risks and benefits were explained. Information sheets were made available to the parents. Written consent was obtained from the parents of participating children.

On average, seven to nine children participated at each playground. This number varied because it was determined by an uncontrollable factor, the number of children actually playing at each playground on a given sampling day. The only exclusion criterion was the absence of a parent’s consent. Study protocols were approved by the University of Alberta Health Research Ethics Board.

After playing in the playgrounds, the participating children provided hand-washing samples. The hand-washing sampling consisted of collecting the washings of children’s hands, after playing in the playgrounds, with deionized water. Sterile medium-sized Ziploc bags (18 × 20 cm; Johnson and Son Ltd., Brantford, ON, Canada) were filled with 150 mL of deionized water at the beginning of every day of sampling. At each playground, once children had finished playing, their hands were rinsed for 1 min in the Ziploc bags containing deionized water. The age of each child and the length of time the child had played in the playground were recorded to correspond with the correct hand-washing sample.

The hand-washing samples were then brought back to the laboratory, where they were carefully poured into sterile polystyrene bottles. Each bag was rinsed with 80 mL of deionized water, and the rinse solution was added to the corresponding sample in the polystyrene bottle. The samples (total of 230 mL) were stored at 4°C until analysis. A control sample was also prepared in the same manner for every day of sampling, in which no hands were washed, but all other steps were followed.

### Determination of arsenic in hand-washing samples.

We analyzed the washings collected for every child in all of the 16 playgrounds together on the same day for total arsenic concentrations. Because the hand washing contained residual sand from children’s hands, the concentrations of arsenic in the solution and the sand were determined separately after filtration. Hand-washing samples were filtered using Whatman glass filters with 1.2-μm pore size (Whatman International Ltd., Maidstone, UK). The sand collected on the filters was dried at 140°C and weighed. This provided direct measurements of the amount of sand on children’s hands.

We collected the filtrate for the analysis of soluble arsenic in the washing. To 10 mL of filtered samples we added 100 μL of concentrated HNO_3_ to give an overall concentration of 1% nitric acid. Concentrations of total arsenic (micrograms per liter) in each hand-washing sample were determined from triplicate analyses. Arsenic concentration multiplied by the volume of hand-washing solution (230 mL) provided the total amount (micrograms) of soluble arsenic on children’s hands.

We quantified the arsenic using an inductively coupled plasma-mass spectrometer (6100DRC Plus; PerkinElmer Sciex, Concord, ON, Canada). The standard liquid sample introduction system consisted of a Meinhard nebulizer coupled to a cyclonic spray chamber (Glass Expansion, West Melbourne, Australia). We used an ASX-500 autosampler (CETAC Technologies Inc., Omaha, NE, USA) to introduce the samples. The flow rate for sample introduction was set to 0.8 mL/min. The radio-frequency power was 1100 W. The argon gas flow rates were 15 L/min (plasma gas), 1.2 L/min (auxiliary gas), and 0.9 L/min (nebulizer gas), respectively. Rhodium (5 μg/L) was used as an internal standard. Calibration of the inductively coupled plasma mass spectrometer (ICPMS) using eight arsenic concentrations (0, 0.4, 0.8, 1.2, 1.6, 2.0, 5.0, and 10.0 μg/L) was carried out every 50 samples, and standard reference material (SRM) 1640 [National Institute of Standards and Technology (NIST), Gaithersberg, MD, USA], trace element in natural water (after 10-fold dilution) was analyzed every 10 samples as a quality control. The measured values of arsenic in the SRM were 24.1 ± 1.8 μg/L from 14 repeat analyses spaced over 2 days. This is in good agreement with the certified value (26.67 ± 0.41 μg/L) of the SRM.

### Playground sand/soil samples.

Three composite sand/soil samples were collected from each playground on the same day when the children’s hand-washing samples were obtained. They were collected from the under-deck areas, the areas in which children frequently played, and the areas away from any playground structures. Sand and soil were taken from these areas to a depth of 0–6 inches, mixed, and placed in separate clean glass containers. The exact locations of sampling were marked on a detailed plan of each playground, as was the time of sampling. In addition, two playgrounds (G and R) containing CCA-treated wood structures were extensively sampled, with 24 samples collected at 0- to 6-inch depth from various locations of these playgrounds, particularly the areas frequently accessed by children.

### Determination of arsenic in playground soil/sand samples.

The level of arsenic in the sand/soil samples was determined by EnviroTest Laboratories (Edmonton, AB, Canada) according to its Standard Operating Procedures. Briefly, we followed U.S. EPA SW-846 method 3050B (U.S. EPA 1996) for the acid digestion of the sand/soil samples. A representative 1–2 g sample was digested in nitric acid and hydrogen peroxide. The digest was then refluxed with nitric acid until all solid material was completely dissolved. The digest was diluted with deionized water, and the final solution contained 5% nitric acid.

We determined the arsenic concentration in the digest using an Elan 6000 ICPMS (PerkinElmer Sciex), following U.S. EPA method 6020 (U.S. EPA 1994). Included in the ICPMS analytical runs were the following quality control samples: calibration verification (every 10 samples), reagent blanks (every 10 samples), method blanks (one per batch), matrix spikes (10% of samples), sample duplicates (10% of samples), and NIST SRM 2709 (one per batch).

### Statistical analysis.

Statistical analysis was performed using SPSS (version 11.5; SPSS Inc., Chicago, IL, USA). Data are expressed as mean ± SD. The total amount of soluble arsenic on children’s hands and the amount of arsenic in soil/sand values were compared between CCA and non-CCA playgrounds using two-independent samples *t*-test. The age of the children, the length of time the children played, and the concentration of arsenic in the sand/soil from the playgrounds were also compared by using *t*-test. The Pearson correlation coefficient was computed between all the continuous measurements. In multivariate analysis, arsenic on children’s hands was compared between CCA and non-CCA playgrounds after controlling for all the other variables using a general linear model. A *p*-value < 0.05 was considered statistically significant.

### Risk evaluation.

Risk calculation followed the U.S. EPA’s risk assessment framework (U.S. EPA 2001 U.S. EPA 2002b). Ingestion was considered the main route of exposure. Exposure to arsenic by dermal absorption and inhalation was considered negligible (Bernstam et al. 2002; Wester et al. 2004). We used the measured values of arsenic on children’s hands for risk estimation. For comparison, we also used the amount of incidental ingestion of soil arsenic, estimated from the amount of sand ingested per day and the concentration of arsenic in the sand. The latter followed the usual assumption that the amount of sand ingested by children (2–6 years of age) is 50% of the total amount of sand (100 mg) on children’s hands.

## Results

### Demographics of the participating children.

**Figure 1:**
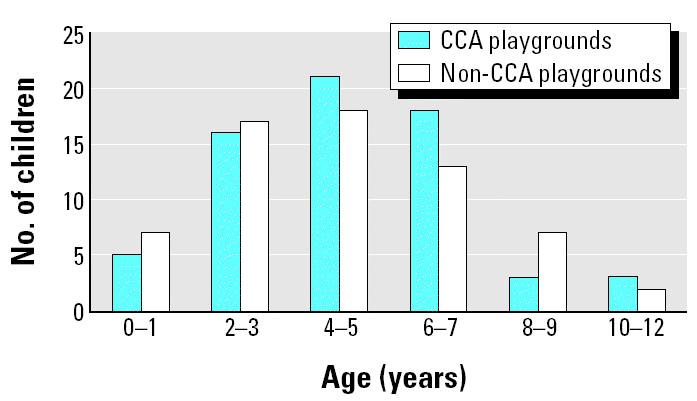
Distribution of the ages of children who provided hand-washing samples.

One hundred thirty children participated in this study, of whom 66 (50.8%) were from CCA playgrounds and 64 (49.2%) were from the non-CCA playgrounds. Seventy participating children (53.8%) were boys, and 60 were girls (46.2%). The ages (mean ± SD) of the participating children were 4.7 ± 2.5 years for the CCA playgrounds and 4.8 ± 2.4 for the non-CCA playgrounds (Figure 1). There was no significant difference in the children’s ages between the two groups (*p* = 0.82).

We selected all children at each playground whose parents were able to provide written consent. Most children (> 80%) playing at each playground during the sampling period of 3–5 hr participated in the study. Thus, on average there were seven to nine participating children from each playground. A total number of 130 participating children is reasonable considering that 42% of children 2–6 years of age spend < 3 hr/day outdoors and that 80% of children under the age of 11 years spent ≤1 hr each day playing outdoors on sand, gravel, dirt, or grass (U.S. EPA 2002b).

### Length of play time in the playgrounds.

**Figure 2:**
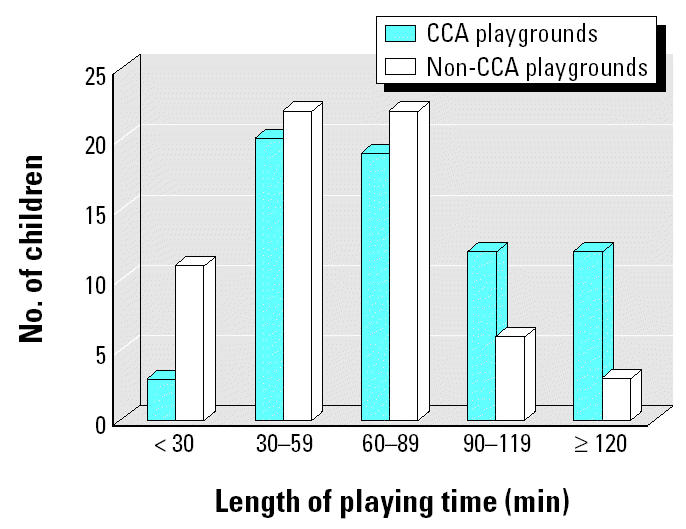
Distribution of the length of time children played in the CCA playgrounds and non-CCA playgrounds.

Figure 2 compares the length of time children played in the playground before hand-washing samples were obtained from the children. The mean length of play time was 74.4 ± 45.7 min (median, 60 min; range, < 30–240 min) for the CCA playgrounds and 49.4 ± 27.6 min (median, 45 min; range, < 30–120 min) for the non-CCA playgrounds. Although the average length of play between the two groups was different, this is mainly driven by a few children (*n* = 8) who played > 120 min in the CCA playgrounds compared with the non-CCA playgrounds, where three children played for 120 min and no children played > 120 min.

### Concentration of arsenic in the sand/soil from the playgrounds.

Table 1 shows the concentration of arsenic in sand/soil samples collected from the 16 playgrounds. The values of arsenic concentration in sand/soil (mean ± SD) were 3.3 ± 1.7 (median, 2.9; range, 0.8–7.4) for the CCA playgrounds and 1.9 ± 1.2 (median, 1.8; range, 0.5–5.3) for the non-CCA playgrounds. Although the concentrations of arsenic in the samples from the different playgrounds vary, there is no significant difference between the two groups (*p* = 0.07).

To examine possible heterogeneity of arsenic concentration in sand/soil samples from the playgrounds, we conducted extensive multiple sampling from two CCA playgrounds (G and R). We collected 24 samples from each of these playgrounds and analyzed them for arsenic concentration. The values (mean ± SD) of arsenic concentration were 3.5 ± 1.4 (median, 3.1; range, 1.3–6.0) for playground G and 3.5 ± 1.5 (median, 2.9; range, 1.7–7.4) for playground R. Both of these playgrounds contained CCA-treated wood structures.

### Amount of soluble arsenic in the hand washing.

Table 2 shows the amount of soluble arsenic in hand washing of children playing in the 16 playgrounds. The hand washing was filtered to remove residual sand and the filtrate was directly analyzed for soluble arsenic present in the hand washing. Thus, these results represent the amount of soluble arsenic on children’s hands that were washed with 150 mL water. The overall values were 501 ± 512 ng (median, 398 ng; range, 8–3,536 ng) for the CCA playgrounds and 95 ± 70 ng (median, 72 ng; range, 11–407 ng) for the non-CCA playgrounds. The levels of arsenic on children’s hands were significantly (*p* < 0.001) higher for children playing in the CCA playgrounds compared with those in the non-CCA playgrounds.

### Amount of arsenic in the sand residue collected in the hand washing.

Table 3 shows the amount of sand collected from children’s hand washing. The fine sand particles were collected in the hand washing and were filtered, dried, and weighed. The amount of sand collected from children’s hands in dry weight was 22.0 ± 19.1 mg (median, 16.4 mg; range, 0.8–95.8 mg) for the CCA playgrounds and 25.2 ± 23.3 mg (median, 16.6 mg; range, 3.7–116.2 mg) for the non-CCA playgrounds. There was no significant difference between the two groups (*p* = 0.23) regarding the amount of sand on the children’s hands. This is not surprising because of the similar age distributions (thus similar distribution of the size of hands) and the similar dry weather conditions during the sampling (thus similar adsorption of sand).

### Total amount of arsenic in the hand washing.

Table 4 summarizes the total amount of arsenic in the hand washing. The values correspond to the sum of soluble arsenic and the arsenic in the sand residue collected in the hand washing for each child. The overall values were 561 ± 552 ng (median, 416 ng; range, 8–3,865 ng) for the CCA playgrounds and 143 ± 95 ng (median, 124 ng; range, 23–475 ng) for the non-CCA playgrounds. The levels of total arsenic on children’s hands were significantly (*p* < 0.001) higher for children playing in the CCA playgrounds than for those in the non-CCA playgrounds. This difference is primarily driven by the soluble arsenic (Table 2).

### Additional statistical analysis.

**Figure 3:**
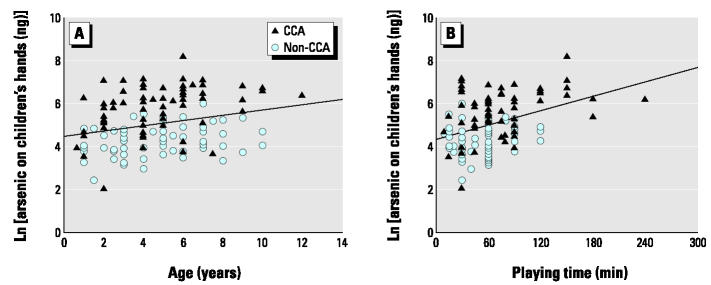
Plots showing weak correlation of arsenic concentration from CCA and non-CCA playgrounds on children’s hands with (*A*) children’s age (*r* = 0.24) and (*B*) the length of playing time (*r* = 0.33). The arsenic concentration was logarithmically transformed. The results for soluble arsenic on the hands of 66 children who played in CCA playgrounds and 64 children who played in non-CCA playgrounds are included.

There is no difference between boys and girls with regard to the amount of arsenic in their hand washing. Figure 3 shows that the amount of arsenic seems to increase with increasing age of children. However, there is no clear correlation (*r* = 0.24) between the children’s age and the amount of arsenic on their hands. Similarly, there is a very weak correlation (*r* = 0.33) between the length of play time and the amount of arsenic on their hands (Figure 3).

In multivariate analysis, the amount of arsenic on children’s hands remains significantly (*p* < 0.001) higher for those children who played in the CCA playgrounds than for those who played in the non-CCA playgrounds, even after controlling for the age of children and length of play time.

## Discussion

The playgrounds were selected to represent the geographic locations of the entire city. The age and the manufacturers of the playgrounds were matched between the two groups. Sampling from the CCA and non-CCA playgrounds was carried out on alternate days. The weather conditions during sample collection were similar between the two groups. Therefore, with these variables controlled, we are able to examine any other differences between the playgrounds with or without the CCA-treated wood structures.

The ages of children and the length of their playing time in both groups of playgrounds were not controlled by design because we wanted to include as many participating children as possible. All children playing in the playgrounds during the time of our visit were approached, and those with their parents’ written consent participated in the study. The ages of children and the length of their playing time in both groups of playgrounds were not significantly different (Figures 1 and 2).

Results for arsenic in the sand/soil samples from the playgrounds show that there is no significant difference between the CCA and non-CCA playgrounds. The concentrations of arsenic in these samples from both types of playgrounds (3.3 ± 1.7 and 1.9 ± 1.2 mg/kg, respectively; Table 1) are below the Canadian guideline value of 12 mg/kg, established by the Canadian Council of Ministers of the Environment (CCME) National Contaminated Sites Remediation Program for all land use (residential/parkland) in Canada (CCME 1995). The guideline value was based on an estimated lifetime incremental risk of 10^−6^ and a soil ingestion rate of 20 mg/day. We found 22 ± 19 and 25 ± 23 mg sand from the hands of children playing in the CCA and non-CCA playgrounds, respectively (Table 3). Assuming that all the soil on children’s hands is ingested, the measured amount and the estimated values are similar.

The most important and significant difference between the playgrounds with or without the CCA-treated wood structures is the levels of arsenic found in the washings of children’s hands. The total amount of arsenic (Table 4), including both water-soluble arsenic in the washing water (Table 2) and arsenic in the sand collected from children’s hands (Table 3), was significantly higher for the CCA group (561 ± 552 ng) than for the non-CCA group (143 ± 95 ng). This difference was dominated by the soluble arsenic in the hand-washing water, which was approximately 5-fold higher in the CCA group (501 ± 512 ng) than in the non-CCA group (95 ± 70 ng; Table 2). Young children (2–6 years of age) have, on average, a hand-to-mouth frequency of 8–10/hr (Reed et al. 1999; Tulve et al. 2002). Young children putting hands and/or fingers in their mouths could lead to ingestion of arsenic that is on their hands (Tulve et al. 2002). Therefore, CCA-treated wood structures in playgrounds could potentially contribute to a higher exposure to arsenic by children playing in these playgrounds.

It is interesting to note that although there is a significantly higher concentration of arsenic on the hands of children playing in the CCA playgrounds, there is no significant difference in the concentration of arsenic in the sand/soil. A most likely reason responsible for this difference is that children in the CCA playgrounds may be in contact with CCA-treated wood structures directly. It has been found that arsenic from the CCA-treated wood can be transferred onto the hands when rubbing hands against the wood surface [California Department of Health Services (CDHS) 1987]. This is consistent with the results of analysis of swipe samples of CCA-treated wood carried out by others (Osmose Research Division 1983) and by us (data not shown). A subsequent investigation comparing hand washing from children in contact with CCA-treated wood and the same group of children playing with sand in CCA playgrounds suggests that direct contact of hands with CCA-treated wood is a major contributor to the increases in concentration of arsenic on children’s hands (Lu X and Le XC, unpublished data).

The maximum amount of arsenic on children’s hands was < 4 μg (Table 4). This is equivalent to 0.22 μg/kg body weight using a default body weight value of 17.8 kg for young children (2–6 years of age). With a safe conservative assumption that all the arsenic on children’s hands is ingested, the measured value is below the estimated average daily intake of inorganic arsenic from water and food by Canadian children, which is approximately 0.6 μg/kg body weight (CCME 1995). The average daily dietary ingestion of total arsenic was estimated to be 38 μg (15 μg for children 1–4 years of age) for Canada (Dabeka et al. 1993), 62 μg for the United States (Gartrell et al. 1988), 89 μg for the United Kingdom (Food Additives and Contaminants Committee 1984), 55 μg for New Zealand (Dick et al. 1978), and 160–280 μg for Japan (Tsuda et al. 1995). A range of arsenic species that have different toxicities may be present in food (Le et al. 2004). Estimated daily dietary intake of inorganic arsenic was 8.3–14 μg in the United States (Yost et al. 1998), 4.8–12.7 μg in Canada (Yost et al. 1998), and 15–211 μg in Taiwan (Schoof et al. 1998).

It is important to point out to the general public that arsenic is naturally present in the soil and sand regardless of whether the playgrounds contain CCA-treated wood structures. An important approach to reducing children’s exposure to arsenic is to wash hands after playing, particularly after contact with CCA-treated wood. We have measured arsenic in sequential hand washings and found that the most arsenic was present in the first hand washing (data not shown). This confirms that hand washing is effective in removing arsenic from hands.

## Conclusions

Children playing in playgrounds constructed with CCA-treated wood have approximately five times more arsenic on their hands than do those playing in playgrounds that do not have CCA-treated wood structures. The higher values of arsenic on the hands of children playing in the CCA playgrounds are probably caused by direct contact with CCA-treated wood. Most of the arsenic on children’s hands is water soluble and is readily washed off with water. We recommend that children wash their hands after playing to reduce their potential exposure to arsenic.

The concentrations of arsenic in soil/sand samples from both CCA and non-CCA playgrounds were below the Canadian guideline levels. The maximum amount of arsenic on children’s hands from the entire group of study participants was < 4 μg. This amount is lower than the average daily intake of arsenic from water and food.

This study provides direct measurements of the amount of arsenic on children’s hands. The results—along with other information, such as the frequency and habit of hand-to-mouth activity, efficiency of transfer of arsenic from hands to mouth, and repeated contact of hands with CCA-treated wood surface after hand-to-mouth activity—are useful for assessing children’s exposure to arsenic.

## Figures and Tables

**Table 1:** Concentration of arsenic (mg/kg) in sand/soil samples collected from 16 playgrounds.

Playground	Mean ± SD	Median	Range
CCA playgrounds			
A	3.9 ± 2.6	3.8	1.4–6.5
C	1.2 ± 0.4	1.3	0.8–1.5
D	2.6 ± 2.0	1.6	1.3–4.9
F	3.4 ± 3.3	1.5	1.5–7.3
G	3.5 ± 1.4	3.1	1.3–6.0
I	2.2 ± 1.3	1.5	1.4–3.7
N	2.7 ± 2.6	1.6	0.8–5.6
R	3.5 ± 1.5	2.9	1.7–7.4
Overall	3.3 ± 1.7	2.9	0.8–7.4
Non-CCA playgrounds			
B	1.8 ± 0.1	1.7	1.7–1.9
E	1.9 ± 0.1	1.9	1.8–2.0
H	3.3 ± 1.8	2.9	1.8–5.3
J	0.6 ± 0.1	0.6	0.5–0.7
K	2.2 ± 0.6	2.3	1.6–2.8
L	1.1 ± 0.8	0.7	0.5–2.0
M	1.2 ± 0.4	1.3	0.8–1.6
O	3.0 ± 1.3	2.2	2.2–4.5
Overall	1.9 ± 1.2	1.8	0.5–5.3

**Table 2:** Amount of water-soluble arsenic (ng) in hand washing from children playing in the 16 playgrounds.

Playground	Mean ± SD	Median	Range
CCA playgrounds			
C	272 ± 152	330	50–479
D	956 ± 247	871	570–1,263
F	167 ± 84	167	108–226
G	670 ± 300	691	185–1,126
I	359 ± 223	271	84–784
N	196 ± 157	147	8–500
R	987 ± 1,161	485	163–3,516
Overall	501 ± 512	398	8–3,536
Non-CCA playgrounds			
E	82 ± 27	79	51–113
H	68 ± 36	60	26–138
J	60 ± 44	51	23–136
K	123 ± 51	113	46–225
L	38 ± 13	39	19–56
M	215 ± 95	193	129–407
O	61 ± 37	58	11–114
Overall	95 ± 70	72	11–407

**Table 3:** Amounts of sand and sand arsenic collected in hand washing from children playing in the 16 playgrounds.

	Sand (mg)			Arsenic (ng)		
Playground	Mean ± SD	Median	Range	Mean ± SD	Median	Range
CCA playgrounds						
A	24.2 ± 24.9	13.9	5.0–77.7	70 ± 72	40	15–225
C	21.9 ± 23.0	14.0	5.2–76.5	26 ± 28	17	6–92
D	26.7 ± 10.3	26.3	15.0–42.8	69 ± 27	68	39–111
F	20.4 ± 21.8	20.4	5.0–35.8	70 ± 75	70	17–123
G	31.7 ± 20.4	29.5	3.3–67.7	110 ± 71	103	11–235
I	18.9 ± 11.1	14.5	6.6–38.2	42 ± 24	32	15–84
N	11.4 ± 10.1	7.7	0.8–38.3	30 ± 27	20	2–102
R	29.7 ± 31.0	16.4	10.8–95.8	102 ± 106	56	37–329
Overall	22.0 ± 19.1	16.4	0.8–95.8	60 ± 60	43	2–329
Non-CCA playgrounds						
B	40.5 ± 40.3	28.7	7.2–116.2	72 ± 71	51	13–205
E	21.3 ± 13.2	21.2	6.5–37.3	41 ± 25	40	12–71
H	15.1 ± 9.2	13.0	5.7–38.2	50 ± 31	43	19–127
J	27.5 ± 32.9	13.3	9.1–86.1	17 ± 20	8	5–52
K	25.3 ± 10.8	24.8	10.2–45.7	56 ± 23	55	23–102
L	9.0 ± 2.9	9.5	3.7–11.7	10 ± 3	10	4–12
M	38.2 ± 23.9	45.3	10.8–73.1	47 ± 30	56	13–90
O	23.8 ± 22.6	15.3	3.8–70.2	71 ± 67	46	11–208
Overall	25.2 ± 23.3	16.6	3.7–116.2	49 ± 44	42	4–208

**Table 4:** Amount of total arsenic (ng) in hand washing from children playing in the 16 playgrounds.

Playground	Mean ± SD	Median	Range
CCA playgrounds			
A	587 ± 385	555	48–1,260
C	298 ± 172	356	57–571
D	1,025 ± 252	947	646–1,325
F	237 ± 158	237	125–349
G	780 ± 350	850	196–1,295
I	400 ± 229	297	168–855
N	225 ± 170	204	8–563
R	1,089 ± 1,256	561	219–3,865
Overall	561 ± 552	416	8–3,865
Non-CCA playgrounds			
B	163 ± 134	102	57–420
E	122 ± 40	134	74–171
H	118 ± 55	111	48–265
J	77 ± 63	59	31–188
K	175 ± 55	163	69–281
L	48 ± 15	49	23–67
M	262 ± 112	250	142–475
O	132 ± 92	122	23–277
Overall	143 ± 95	124	23–475
